# Membrane-Mediated Oligomerization of G Protein Coupled Receptors and Its Implications for GPCR Function

**DOI:** 10.3389/fphys.2016.00494

**Published:** 2016-10-25

**Authors:** Stefan Gahbauer, Rainer A. Böckmann

**Affiliations:** Computational Biology, Department of Biology, Friedrich-Alexander University of Erlangen-NürnbergErlangen, Germany

**Keywords:** GPCR, dimerization, oligomerization, membrane, cholesterol, palmitoylation, FRET, molecular dynamics simulation

## Abstract

The dimerization or even oligomerization of G protein coupled receptors (GPCRs) causes ongoing, controversial debates about its functional role and the coupled biophysical, biochemical or biomedical implications. A continously growing number of studies hints to a relation between oligomerization and function of GPCRs and strengthens the assumption that receptor assembly plays a key role in the regulation of protein function. Additionally, progress in the structural analysis of GPCR-G protein and GPCR-ligand interactions allows to distinguish between actively functional and non-signaling complexes. Recent findings further suggest that the surrounding membrane, i.e., its lipid composition may modulate the preferred dimerization interface and as a result the abundance of distinct dimeric conformations. In this review, the association of GPCRs and the role of the membrane in oligomerization will be discussed. An overview of the different reported oligomeric interfaces is provided and their capability for signaling discussed. The currently available data is summarized with regard to the formation of GPCR oligomers, their structures and dependency on the membrane microenvironment as well as the coupling of oligomerization to receptor function.

## 1. Introduction

G protein coupled receptors (GPCRs) form the largest and most diverse group of transmembrane proteins and are considered key players involved in numerous processes, including in particular the communication between cells. The membrane-embedded portion of all GPCRs is built by seven transmembrane helices (TM1-TM7). Upon activation they couple to a G protein at the intracellular, carboxyl-terminal part. The activation-inducing ligand is received from the extracellular domain of the receptor. For both, ligands and G proteins, GPCRs typically show a high plasticity with regard to engaging with different types of interaction partners, hence a single GPCR can be involved in many different signaling pathways. Even though GPCRs have been studied for several decades, certain aspects of their function still lack a complete characterization. Until the early 2000s, GPCRs were considered as functional monomeric units. However, a continuously growing number of studies reported the presence of homo- or heterodimers or even higher-order oligomers throughout the vast family of these receptors (Angers et al., 2002). The importance of receptor assembly is still debated: On the one hand, different studies present evidence that dimerization is required prior of signaling, especially for the group of class C GPCRs (Kniazeff et al., 2011). On the other hand, for the overall less investigated class B GPCRs, functional monomers were shown by actively disrupting dimerization by mutations (Pioszak and Xu, 2008). Still, there is rising evidence that these receptor types form also functional dimers (Ng et al., 2012).

Similar findings were reported for the medically most relevant and largest group of class A GPCRs: Evidence has been collected for both, functional monomers as well as functional dimers or oligomers. For example signaling monomers have been reported for rhodopsin (Bayburt et al., 2007) and the β_2_-adrenergic receptor (Whorton et al., 2007), while the same receptors have been observed in dimeric configurations in crystal structures (Palczewski et al., 2000; Cherezov et al., 2007). Additionally, other experiments also hint to functional dimers of these receptors (Angers et al., 2000; Jastrzebska et al., 2015). In general, the ongoing development and improvement of membrane protein crystallography allowed to resolve several crystal structures of GPCRs (Ghosh et al., 2015), frequently with the receptors in stable dimeric conformations (Rosenbaum et al., 2009; Katritsch et al., 2012). However, crystal dimer configurations may not necessarily reflect native dimer configurations, since the crystallization may require sequence modifications and in particular since the crystal environment differs considerably from the membrane environment *in vivo*. In addition to the growing number of structures, more and more computational methods were employed to unveil the dynamics of GPCR multimerization (Meng et al., 2014; Kaczor et al., 2015). The functionality of GPCR dimers was further validated by advanced fluorescence methods such as Förster resonance energy transfer (FRET), bioluminescence resonance energy transfer (BRET), or fluorescence correlation spectroscopy (FCS) experiments, which allow to measure signaling and aggregation of GPCRs simultaneously (Goddard and Watts, 2012a; Kasai and Kusumi, 2014; Vischer et al., 2015).

Based on the continuously growing number of studies indicating class A GPCR assembly as a part of proper signaling, the process of dimer formation and differences in dimer interfaces among different GPCRs shifted into the focus. Recent cross-linking studies revealed that active GPCRs may form dimer interfaces that differ from that of their inactive conformation (Guo et al., 2005; Mancia et al., 2008). These intriguing shifts in GPCR dimerization interfaces suggest its involvement in the regulation of GPCR activation and signaling strength.

Structural studies revealed that GPCRs share a typical conformational change upon activation. This configurational transition mainly involves an outwards movement of TM6, opening up the intracellular part of the TM helix bundle in order to enable G protein coupling (Kobilka, 2007; Kimata et al., 2015). In addition to this commonly observed conformational change, functional selectivity of the serotonin 5-Hydroxytryptamine(2A) (5-HT_2*A*_) receptor was assigned to smaller yet specific ligand-dependent conformational changes of the intracellular loop 2 (connecting TM3 and TM4) (Perez-Aguilar et al., 2014). These observations allow to hypothesize that the modulation of dimeric or oligomeric interfaces may contribute to the functional selectivity by inducing or hindering specific conformational changes. For instance, involvement of TM6 at the dimer interface, may possibly result in conformational trapping of TM6 in an activation-incompetent conformation (Cordomí et al., 2015; Vafabakhsh et al., 2015). Vice versa, active receptor dimer configurations will likely require a conformationally flexible TM6 helix, i.e., a dimer interface without TM6 (e.g., a symmetric interface around TM1 and TM4). Such a dimer-specific activity and functional selectivity could offer new opportunities to target GPCR function with medical drugs (Hipser et al., 2010).

Furthermore, an increasing number of studies suggests that dimerization and signaling of GPCRs are modulated by the surrounding membrane. This review will focus on how different types of lipids and other membrane components may influence dimerization patterns of GPCRs and thereby possibly regulate function and signaling. There are two possible paths for how lipids may influence GPCRs association: by direct binding to the receptor surface, or indirectly by modulating the properties of the surrounding membrane. Here, we review and discuss available information for both mechanisms, as well as commonly employed methods for the study of GPCR oligomerization.

## 2. Methods to analyze GPCR oligomerization

Two frequently used methods to analyze GPCR dimer- or oligomerization are resonance energy transfer (RET) techniques and computational approaches such as molecular dynamics (MD) simulations. In FRET and BRET experiments, the resonance energy transfer between an energy donor and an energy acceptor is used to analyze protein association (Cottet et al., 2012). The efficiency of this non-radiative energy transfer (via long-range dipole-dipole interactions) is inversely proportional to the sixth power of the distance between donor and acceptor (Förster, 1948). In general RET methods have a spatial resolution of 10–100 Å (Sekar and Periasamy, 2003). FRET studies are performed by fusing fluorescent proteins to the C-terminal parts of the GPCRs of interest. The fluorescent proteins are usually variants of the green fluorescent protein (GFP) (Shimomura et al., 1962) that show a certain overlap of their excitation and emission spectra (e.g., CFP and YFP), in order to allow for the absorption of the donor-emitted light by the acceptor.

Due to the fact that the fluorophor proteins consist of approximately 230 amino acids, hence are of comparable size as GPCRs themselves, the receptor stability needs to be tested and possible fluorophor-driven associations between the proteins should be excluded (see e.g., Lohse et al., 2012). As an alternative to the large GFP, small peptides binding extracellular fluorescein were presented that may in many cases reduce steric side effects (Griffin et al., 1998).

**Figure 1:**
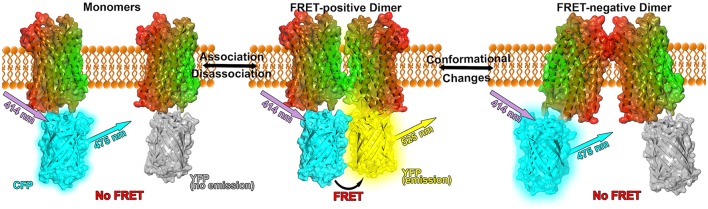
**Model of FRET experiments applied to GPCR dimerization**. The GPCR (here: CXCR4 chemokine receptor, PDB: 3ODU, Wu et al., 2010) is shown in surface and cartoon representation and colored in a lateral gradient from red to green. Fluorescent proteins (PDB: 1YFP, Wachter et al., 2000) are linked to the C-terminal part of the crystal structures. CFP is shown in cyan, unexcited YFP in gray and YFP in its excited state in yellow (Figure inspired by Lohse et al., 2012; Broussard et al., 2013).

If the donor is excited via light waves at its absorbance wavelength and the acceptor is in close vicinity, FRET occurs between the donor and the acceptor resulting in a detectable emission of light from the acceptor (see Figure 1). However, not only the distance but as well the relative orientations between donor and acceptor affect the energy transfer: If the dipoles are oriented perpendicular to each other, no FRET occurs. Only a parallel orientation between donor and acceptor dipoles allows for a resonance energy transfer. Further the efficiency of the energy transfer between FRET proteins is rather small (about 10–40 %), hence only small fluorescence intensities have to be measured which can be quite challenging (Broussard et al., 2013). Therefore, experimental instruments need to be justified carefully and extensive mathematical analysis of the collected signals is required for reliable conclusions. Additionally, the absence of FRET is not equivalent to the absence of protein-protein interactions, the fluorophors might not be in a close enough proximity or their relative dipole orientation might not be properly aligned, i.e., the proteins formed a FRET-negative configuration. Furthermore, the surrounding environment can quench the fluorescence up to a degree that no FRET signals can be detected. On the other hand, the overexpression of fluorescence proteins or high protein concentrations can lead to FRET between non-interacting proteins in close vicinity.

In BRET assays, a bioluminescent protein (e.g., luciferase from *Renilla reniformis*, Rluc) is used as the donor, i.e., no light excitation of the donor is required but a substrate (e.g., coelenterazine h for Rluc) in order to stimulate the bioluminescence. Rluc can serve as a donor with YFP or GFP as the acceptor (Ayoub and Pfleger, 2010). The basic advantages of BRET techniques are the reduced background signal as compared to FRET methods, since the excitation is biochemically triggered instead of light-induced. Additionally, BRET enables to perform kinetical measurements, given that the signals can be detected for up to 30 min (Cottet et al., 2012). A notable drawback of BRET strategies as compared to FRET techniques is that the substrate not only excites the bioluminescent proteins at the cell surface but possibly as well cell-interior proteins.

FRET and BRET techniques are very powerful tools to investigate protein association, however the interpretation of RET efficiencies can be rather challenging because different efficiencies can result from either an increased or decreased number of receptor oligomers or from conformational changes in preexisting complexes (see Figure 1). Nevertheless, conformational changes can be concluded if the maximal RET changes while the RET_50_ value (acceptor/donor ratio resulting in a half maximal RET signal) remains unaffected, indicating that the relative affinity between the acceptor and the donor remained equal (Percherancier et al., 2005; Szidonya et al., 2008).

MD simulations provide atomistic detail of biomolecular processes at high spatial and time resolution and proved extremely useful in the study of GPCR dynamics, GPCR oligomer formation and in the analysis of the influence of the membrane environment on oligomerization (Sabbadin et al., 2014; Sengupta and Chattopadhyay, 2015; Tautermann et al., 2015). In general, MD simulations at atomistic resolution of biomolecular systems are limited to the timescale of hundreds of nanoseconds to a few microseconds. However, protein aggregation occurs on timescales of tens of microseconds. Additionally, membrane systems typically contain more than 100,000 atoms and are therefore computationally rather expensive. Therefore, atomistic MD simulations can hardly be employed in the study GPCR association.

**Figure 2:**
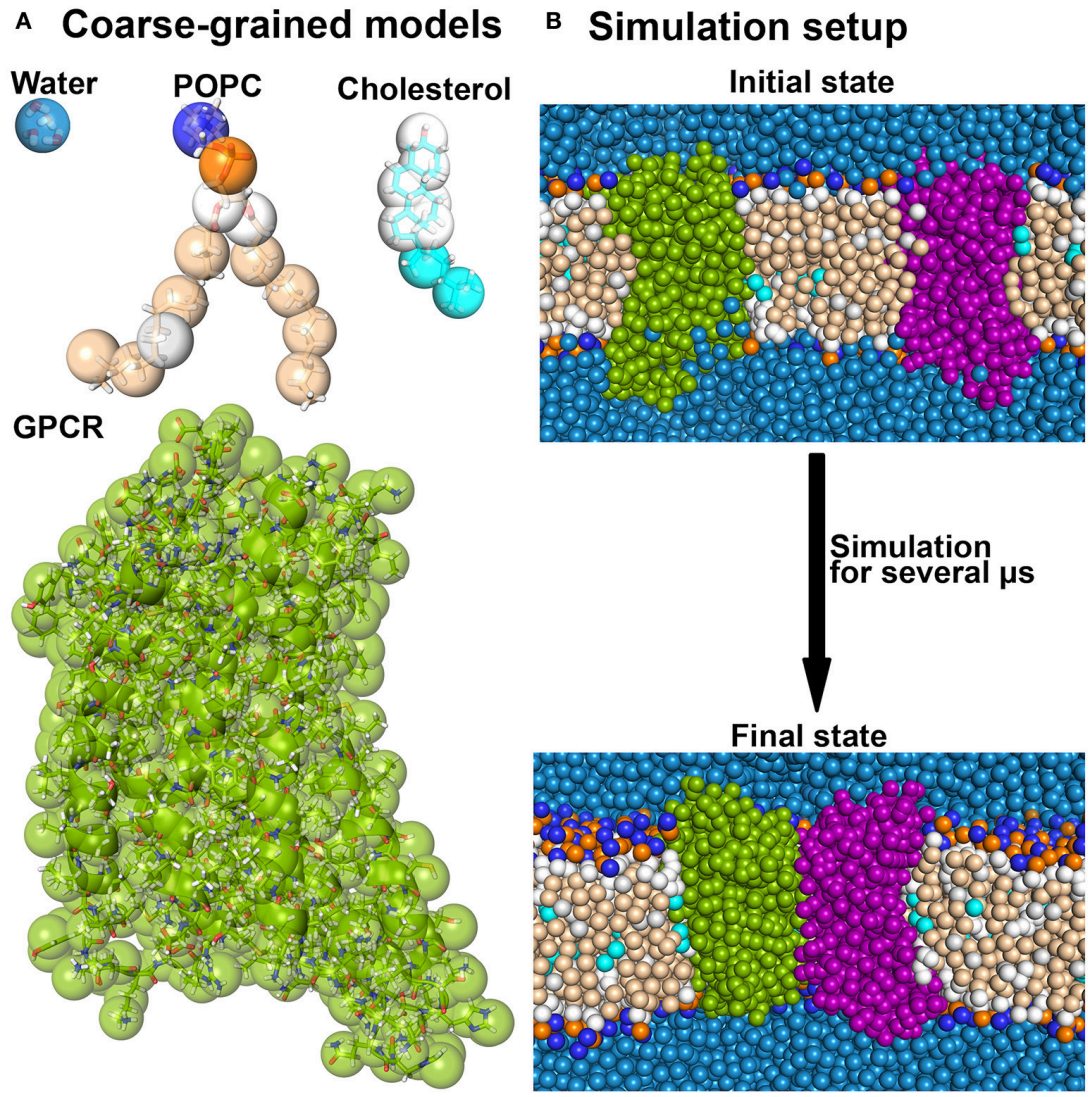
**Coarse-grained MD simulations**. **(A)** Several molecules are shown as overlays between the atomistic and the corresponding Martini coarse-grained models. **(B)** A typical coarse-grained simulation system as used in receptor dimerization is shown before and after the simulation (Pluhackova et al., 2016).

A significant speedup is gained by switching to more coarse-grained resolutions. For example, in the widely used Martini coarse-grained (CG) model, four heavy atoms are grouped together in one superatom (or bead) that reflects the properties of the 4 atoms (Marrink et al., 2004, 2007; Monticelli et al., 2008) (see Figure 2). In this way, the number of degrees of freedom is reduced, high frequency motions of hydrogen atoms are excluded and the diffusion of proteins is increased by a reduced friction between CG beads as compared to atoms. Taken together, the computation time required to study a biomolecular process at CG resolution is by 100–500 times reduced as compared to a corresponding atomistic simulation.

Of course this loss of resolution comes with several drawbacks as compared to atomistic simulations. One important aspect in this regard is the inability of the CG force field to describe conformational changes of proteins, which is due to the simple design of the protein backbone in the Martini force field (Marrink and Tieleman, 2013). In fact, the secondary structure is constrained by an additional force network to keep a preset structure (de Jong et al., 2012). In addition, the reduction of resolution also affects the energy landscape of molecular systems: free solvation energies can be reproduced quite accurately in CG systems as compared to atomistic simulations, however, the decomposition of these free energies into enthalpic and entropic contributions can differ strongly (Marrink and Tieleman, 2013). The reduction of the number of degrees of freedom influences the entropy of the system. In order to reproduce the correct free energy, the enthalpic energies are therefore increased in the Martini model. In the same context, electrostatic interactions between charged particles are described implicitly in order to compensate the reduced number of partial charges and dipoles as compared to atomistic force fields. Consequently, Coulombic interactions in apolar environments are too weak in CG Martini models (Marrink and Tieleman, 2013).

Additionally, interactions between proteins in aqueous solution (Stark et al., 2013; Larisch et al., 2016), membrane-embedded proteins (Prakash et al., 2010; Chavent et al., 2014), as well as between peptides and the membrane interface (Pluhackova et al., 2015) using the standard Martini force field were shown to be artificially enhanced, in particular for the original Martini force field for proteins (Prakash et al., 2010; Stark et al., 2013; Chavent et al., 2014). As a cure, it was shown for the older variant of the Martini force field that a reduction of the Lennard-Jones interactions between protein beads may compensate this overestimation (Stark et al., 2013).

Nonetheless, the Martini forcefield shows good performance for membrane systems, hence it is commonly used to investigate transmembrane proteins on long timescales (Pluhackova et al., 2013; Pluhackova and Böckmann, 2015). GPCR association is usually addressed by simulating a CG system that contains several copies of the receptor, embedded in a membrane bilayer for up to tens of microseconds (Provasi et al., 2015). In this way, different dimeric interfaces can be identified from one simulation. However, the method is hampered by the low dissociation rate, i.e., dimers were not observed to dissociate again on the accessible simulation timescale (Prasanna et al., 2014; Provasi et al., 2015). The obtained statistics from only a few of such simulations is thus quite limited.

A recently developed high-throughput simulation method termed DAFT (Docking Assay For Transmembrane components) offers an automated, extensive sampling of different GPCR dimerization interfaces (Wassenaar et al., 2015b; Pluhackova et al., 2016). In this methodology, several hundreds of CG simulations are performed, each consisting of two copies of a GPCR embedded in an explicit membrane environment and surrounded by water (Wassenaar et al., 2015a). The two proteins are initially placed at a fixed starting distance from each other, but at different starting orientations. Using this ensemble simulation setup, allows to gain statistical insight into GPCR dimerization patterns and how changes in the lipid environment alter the protein association. Despite the aforementioned possible overestimation of protein-protein interactions, the DAFT approach in combination with the Martini force field (de Jong et al., 2012) allowed to determine preferred dimer interfaces for different systems that are in excellent agreement with experiments (Han et al., 2015; Wassenaar et al., 2015b; Pluhackova et al., 2016).

## 3. Compartmentalization of GPCRs in membrane nanodomains

Possible membrane-driven mechanisms that influence the dimerization or oligomerization of GPCRs will sensitively depend on the membrane environment around the receptors. Further, the lipid environment was shown to influence GPCR function and several health disorders during aging were assigned to changes in the membrane composition that altered GPCR signaling (Alemany et al., 2007). The colocalization of GPCRs and other components involved in signal propagation in dynamic membrane nanodomains (sometimes referred to as lipid rafts) has been reported in a vast number of studies (Goddard et al., 2013). These nanodomains are characterized as densely packed, dynamic membrane areas with increased concentrations of glycosphingolipids and cholesterol and have been indicated to play important roles in the sorting and organization of membrane proteins (Villar et al., 2016). Caveolae (“little caves”) show a similar lipid composition, but they additionally contain the protein caveoline on the inner leaflet of the bilayer (Insel et al., 2005). GPCR signaling involves many components like heterotrimeric G proteins (consisting of G_α_, G_β_, and G_γ_ subunits), adenylyl cyclases, channel proteins, phospholipases or GTP exchange factors (Ostrom and Insel, 2004). Signal transduction is mainly performed by specific, physical interactions between these components. However, GPCRs, G proteins and other effector enzymes have been reported to be expressed at low concentrations in cells (Ostrom et al., 2000), thus suggesting a selective compartmentalization of the involved molecules as necessary for efficient signaling. Obviously, the compartmentalization of GPCRs would as well increase the probability for GPCR oligomerization.

Colocalization of GPCRs in caveolae was determined for several GPCRs mainly by comparing fluorescence profiles of caveolin proteins with profiles of marked receptors (Head et al., 2005; Insel et al., 2005). In some cases the receptors colocalized in these domains before any stimulations by an agonist (e.g., β_1_AR and β_2_AR Schwencke et al., 1999; Xiang et al., 2002, serotonin 5-HT_2*A*_ receptor Bhatnagar et al., 2004, or μ-opiod receptor Huang et al., 2007), in other experiments it was observed that the colocalization is agonist-induced (e.g., angiotensin II type 1 receptor Ishizaka et al., 1998, kinin B_1_ receptor Sabourin et al., 2002, or gonadotropin-releasing hormone receptor Pawson et al., 2003). Furthermore, multiprotein complexes involving the β_2_AR were observed in caveolin-rich areas of the membrane (Ianoul et al., 2005).

Non-caveolar nanodomains were also shown to organize GPCRs and regulate their signaling and oligomerization (Xu et al., 2006; Fallahi-Sichani and Linderman, 2009; Villar et al., 2016). A study on the G protein coupled purinergic P2Y12 receptor showed that disruption of functional oligomers, which are incorporated in membrane nanodomains, into non-functional dimers or monomers leads to partitioning of the receptors out of the lipid rafts (Savi et al., 2006). Oligomerization may thus be required for correct localization in membrane nanodomains important for proper signaling.

## 4. Hydrophobic mismatch promotes receptor assembly

**Figure 3:**
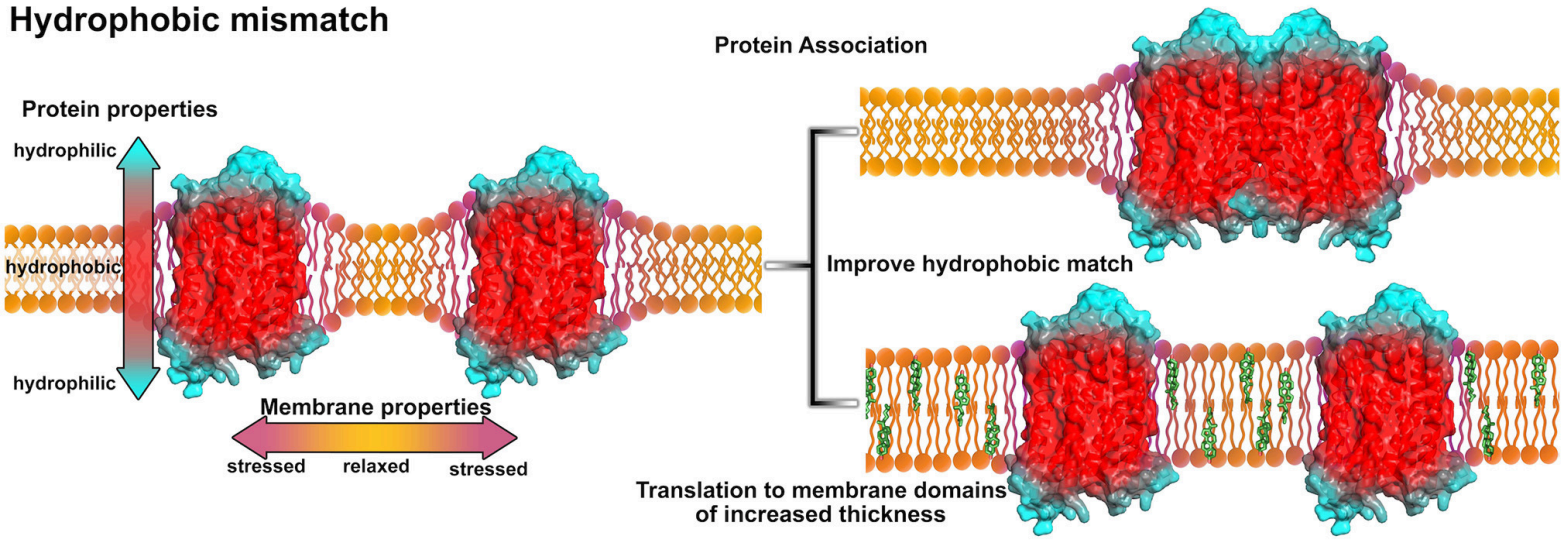
**Influence of the hydrophobic mismatch on membrane-protein systems**. The GPCR (A_2*A*_ adenosine receptor, PDB entry 4EIY, Liu et al., 2012b) is shown in surface representation and colored according to its hypothetical hydrophobicity (red, hydrophobic membrane-embedded portion; blue, hydrophilic, solvent-exposed region). Due to protein insertion, the hydrophobic mismatch induces stress and curvature in the membrane (purple areas in the membrane). In order to reduce membrane stress, the proteins can associate (upper right) or move to membrane areas of increased thickness, e.g., membrane areas with higher cholesterol content (green, lower right). For models on protein-lipid interactions and the hydrophobic match/mismatch please see Kralchevsky et al. (1995) and Mouritsen (2013).

An evident mechanism how membrane properties may regulate GPCR assembly in membrane nanodomains is due to hydrophobic forces coupled to a hydrophobic match or mismatch. The hydrophobic mismatch is defined as the difference between the hydrophobic membrane thickness and the peripheral length of the hydrophobic part of the membrane-spanning protein. Consequently, if the protein's hydrophobic part exceeds the bilayer thickness, oligomerization might reduce the exposed hydrophobic area of the protein (Killian, 1998) (see Figure 3). Using FRET experiments it was shown that the reduction of membrane thickness or the increase of the protein/lipid molar ratio promote rhodopsin association (Botelho et al., 2006). This observation was assumed to be based on the hydrophobic mismatch induced curvature of the membrane at the protein-lipid interface. Association of proteins into protein clusters relieves the curvature free energy (Botelho et al., 2006).

Association of GPCRs to improve the hydrophobic match was additionally analyzed using computational methods to gain molecular insight in this process (Li et al., 2012; Sadiq et al., 2013; Rog and Vattulainen, 2014). Performing coarse-grained MD simulations on systems containing several copies of rhodopsin embedded in membranes of different thickness, it was shown that rhodopsin alters the membrane thickness at the membrane-protein interface (Periole et al., 2007). The shorter the lipid chain length, the more pronounced was the hydrophobic mismatch induced deformation of the bilayer. Local thickening of the membrane was observed near the transmembrane (TM) helices 2, 4, and 7 while thinning was reported near TM1, TM5, TM6, and helix 8 (H8). The most frequently observed dimer conformations were formed by symmetric TM1,2,H8/TM1,2,H8, TM4/TM5, or TM6/TM7 interactions. Overall, oligomerization was enhanced in membranes consisting of lipids with shorter chain length (Periole et al., 2007). Another computational investigation was reported for the adrenergic receptors β_1_AR and β_2_AR. The aim was to predict oligomerization interfaces by analyzing the residual hydrophobic mismatch (RHM, contributions to the energy penalty associated to particular residues) (Mondal et al., 2013). Notably, it was observed that TM1, TM4, and TM5 were most frequently involved in receptor-receptor interactions and showed the highest RHM in monomers which was substantially alleviated in oligomers. Interestingly, in spite of the similarity in sequence and structure, the β_1_AR showed by far the highest RHM for TM1, while TM4 and TM5 revealed a much smaller RHM as compared to β_2_AR. Earlier experiments revealed that β_1_AR indeed mainly dimerized via TM1/TM1 interactions (Calebiro et al., 2013). Inclusion of 10% cholesterol in the membrane overall decreased the RHM, probably by increasing the membrane thickness by inducing an increased order to the lipids' fatty acid chains (de Meyer and Smit, 2009; Mondal et al., 2011).

A few experimental studies addressed the relation between membrane thickness and receptor activity. A more disperse membrane, consisting of diverse lipid types, possibly enabling different membrane domains of different thickness, was shown to facilitate the formation of the active MII state of rhodopsin (Brown, 1994; Botelho et al., 2006). Due to activation, TM6 moves outside of the protomer which overall expands the receptor surface (Liu et al., 2012a) and consequently increases the hydrophobic mismatch. To compensate this effect, the receptor has three options: association with another receptor, translation to a different membrane domain of overall increased thickness, or both. In a more disperse membrane environment, active MII state rhodopsins may likely diffuse to membrane areas with more suitable properties. Additionally, an altered membrane-protein interface due to activation may result in a shift in protonation of a conserved glutamic acid of TM3 in rhodopsin and a thereby stabilized conformation (Sandoval et al., 2016).

Receptor movement to areas of increased membrane thickness was observed for the human δ-opioid receptor using plasmon-waveguide resonance (Alves et al., 2005): The receptor was shown to be preferentially incorporated in POPC-rich domains in the absence of any ligand, while agonist-bound proteins tended to move into sphingomyelin-rich domains of increased thickness (Alves et al., 2005). Possible dimerization of the receptors in both domains was not further investigated. In summary, the hydrophobic mismatch occurs as one of the driving forces in the association and localization of GPCRs in membrane nano- or microdomains.

## 5. Specific membrane components influence GPCR oligomerization and signaling

### 5.1. Cholesterol

One of the most prominent membrane components which shows enlarged concentrations in membrane rafts or nanodomains and was frequently reported to regulate GPCR signaling is cholesterol (Oates and Watts, 2011). In general, cholesterol is considered to increase the membrane thickness by inducing higher order to preferentially saturated lipid tails. Several studies revealed cholesterol as one of the key players in GPCR function, however it should be noted that certain GPCRs have been shown to function as well in membranes free of cholesterol (Oates and Watts, 2011). Furthermore, cholesterol was reported to be important for receptor crystallization (Salom et al., 2013), thus indicating that cholesterol-receptor interactions might be important for the stability of the receptor.

Using dynamic single-molecule force spectroscopy it was shown for the β_2_AR that cholesterol strengthens the interactions between structural segments and thereby stabilizes the receptor kinetically, energetically and mechanically (Zocher et al., 2012). The mechanism behind the stabilization was suggested as either direct binding of cholesterol to the receptor, cholesterol-induced facilitation of receptor oligomerization or as modulation of the bilayer properties. Together with earlier observations that the β_2_AR forms dimers in living cells (Angers et al., 2000; Salahpour et al., 2003) and that the depletion of cholesterol increased coupling of β_2_AR to G_*s*_ proteins (Pontier et al., 2008), this functionally relevant stabilization by cholesterol may not only act on the tertiary structure but moreover on the quaternary structure of the receptors. Previous crystallography studies on the β_2_AR revealed a cholesterol consensus motif (CCM) given as [4.39-4.34(R,K)]−[4.50(W,Y)]−[4.46(I,V,L)]−[2.41(F,Y)] based on the Ballesteros-Weinstein numbering scheme (Hanson et al., 2008). Additional sequence analysis predicted that 21% of class A GPCRs contain this CCM (Hanson et al., 2008), however crystal structures of several of those GPCRs did not show bound cholesterol at this site but on other parts of the receptor (Gimpl, 2016).

In case of the β_2_AR, two cholesterol molecules were bound to the CCM and thereby increased the packing interactions between TM4 and the rest of the helix bundle, yielding an overall increased thermal stability. Additional CG MD simulations on the β_2_AR were performed in order to elucidate the influence of cholesterol on receptor dimerization and it was observed that increasing levels of cholesterol reduce the involvement of TM4 at the dimer interface but enhance the influence of TM1 and TM2 (Prasanna et al., 2014). Due to binding of cholesterol to TM4 (in a similar manner to binding to the CCM) the TM4,5/TM4,5 interface, observed for the receptor in a pure POPC bilayer, was blocked, while increasing levels of cholesterol (9–50%) shifted the dimer configurations over TM1,2/TM4,5 to a symmetric TM1,2/TM1,2 interface.

Modulation of receptor function and organization was also reported for the serotonin 5-HT_1*A*_ receptor. It was observed that the enantiomer of cholesterol (*ent*-cholesterol) could restore ligand binding and receptor signaling after cholesterol-depletion, whereas the diastereomer (*epi*-cholesterol) did not (Jafurulla et al., 2014). *Epi*-cholesterol was shown before to have different biophysical effects on the bilayer due to different tilt angles and phase transition characteristics (Cheetham et al., 1989). The ligand-independent oligomerization of serotonin 5-HT_1*A*_ receptors in living cells was observed with FRET and appeared to be enhanced upon acute cholesterol depletion (Paila et al., 2011). This could be due to reorganization of the receptors induced by the change in membrane properties (e.g., larger hydrophobic mismatch caused by the reduced membrane thickness) or by disrupting specific cholesterol-protein interactions that possibly occupied parts of the protein, thus impeding oligomerization at these parts. Among different GPCRs, including the serotonin 5-HT_1*A*_ receptor, another cholesterol recognition amino acid consensus (CRAC) was identified by performing sequence alignment studies (Jafurulla et al., 2011). The CRAC sequence is defined as [−L/V−(X)_1−5_−Y−(X)_1−5_−R/K−] where (X)_1−5_ denotes between one and five unspecific amino acids, and also the inverse CARC motif [−K/R−(X)_1−5_−Y−(X)_1−5_−L/V−] has been identified as a possible specific cholesterol binding site (Baier et al., 2011). With the aid of CG MD simulations, cholesterol binding to the serotonin 5-HT_1*A*_ receptor was investigated and the highest cholesterol occupancy was obtained for a CRAC motif on TM5 (Sengupta and Chattopadhyay, 2012). A computational model of a symmetric TM4,5/TM4,5 serotonin 5-HT_1*A*_ receptor dimer was generated (Gorinski et al., 2012) based on the obtained molecular model of rhodopsin oligomers from atomic force microscopy maps (Fotiadis et al., 2003). With this model, several amino acids were identified to stabilize the dimer interface which was subsequently tested using FRET experiments on systems containing different types of receptors carrying mutations at the identified residues (Gorinski et al., 2012). Interestingly, mutations of several amino acids reduced the apparent FRET efficiency from 18.9% to about 12%, indicating that the proposed TM4,TM5/TM4,5 dimer is indeed, among other dimer conformations, present in living cells. Together with the aforementioned experiments which revealed a cholesterol-mediated function and activity of the serotonin 5-HT_1*A*_ receptor (Jafurulla et al., 2014), the increased FRET signals upon cholesterol depletion (Paila et al., 2011) and the identified cholesterol binding site on the CRAC motif located on TM5 (Sengupta and Chattopadhyay, 2012), cholesterol appears to regulate receptor activity by occupying the TM5 helix that modulates the involvement of TM5 in dimer conformations.

In case of the chemokine receptor CXCR4, the depletion of cholesterol with either hydroxy-propyl-β-cyclodextrin (Nguyen and Taub, 2002) or methyl-β-cyclodextrin (Wang et al., 2006) abolished CXCR4 signaling by impeding ligand binding. Further FRET studies showed that the absence of cholesterol or blocking the TM4 helix also reduced receptor association (Wang et al., 2006). BRET studies showed that the presence of TM2 and TM4 peptides (derived from TM2 and TM4 of CXCR4) abolished agonist-induced rearrangement in preexisting CXCR4 homodimers and further blocked receptor signaling (Percherancier et al., 2005). TM6 and TM7 petides did not impede conformational changes upon ligand binding, whereas receptor signaling was still reduced (Percherancier et al., 2005). In addition, oxidation of membrane cholesterol to 4-cholesten-3-one catalyzed by the cholesterol oxidase also inhibited binding of an agonist and thus led to reduced signaling of the chemokine receptors CXCR4 and CCR5 (Nguyen and Taub, 2003). These observations indicate the specific requirement of membrane cholesterol for proper receptor function where the sterol may either specifically bind to the protein or alter the membrane properties in a specific manner. In order to shed light on the interplay of cholesterol and CXCR4, CG MD simulations were performed on the CXCR4 crystal structure (Wu et al., 2010) with the goal to investigate the receptor's cholesterol-conditioned homodimerization (Pluhackova et al., 2016). The main findings are that while the most frequently observed dimer interface in pure POPC membranes, namely TM1/TM5-7, is strongly impeded in presence of cholesterol due to specific binding of cholesterol to a groove between TM1 and TM7, the symmetric TM3,4/TM3,4 interface was enabled first by intercalation of cholesterol molecules between the protomers. In orchestra with the previously reported experimental studies where cholesterol depletion and TM4 blocking was shown to reduce CXCR4 signaling, the cholesterol-induced TM3,4/TM3,4 dimer appears as an intriguing candidate for an active conformation. On the other hand, the TM1/TM5-7 interface, highly present in cholesterol-free membranes, might show an inactive dimer based on the fact, that TM6 is directly involved and thereby possibly trapped at the dimer interface (Pluhackova et al., 2016).

For the oxytocin receptor (class A GPCR) it was shown that the presence of cholesterol increases receptor stability and the affinity for agonist or antagonist binding (Klein et al., 1995; Muth et al., 2011). This stabilization was further suggested as being highly specific, given that structure-activity analyses of several cholesterol analogs showed reduced stabilization (Gimpl and Fahrenholz, 2002). *In vivo* oxytocin receptor oligomers have been observed using BRET and it was further shown that a treatment with oxytocin weakens the observed BRET signals (Devost and Zingg, 2004). These observations could hint to a mechanism where cholesterol either specifically alters the membrane properties or binds to the oxytocin receptor and thereby facilitates receptor oligomerization which enhances the activity of the receptor. Ligand-binding may either modulate the oligomeric interface or disrupt protein complexes due to induced conformational changes in the receptor protomers.

**Figure 4:**
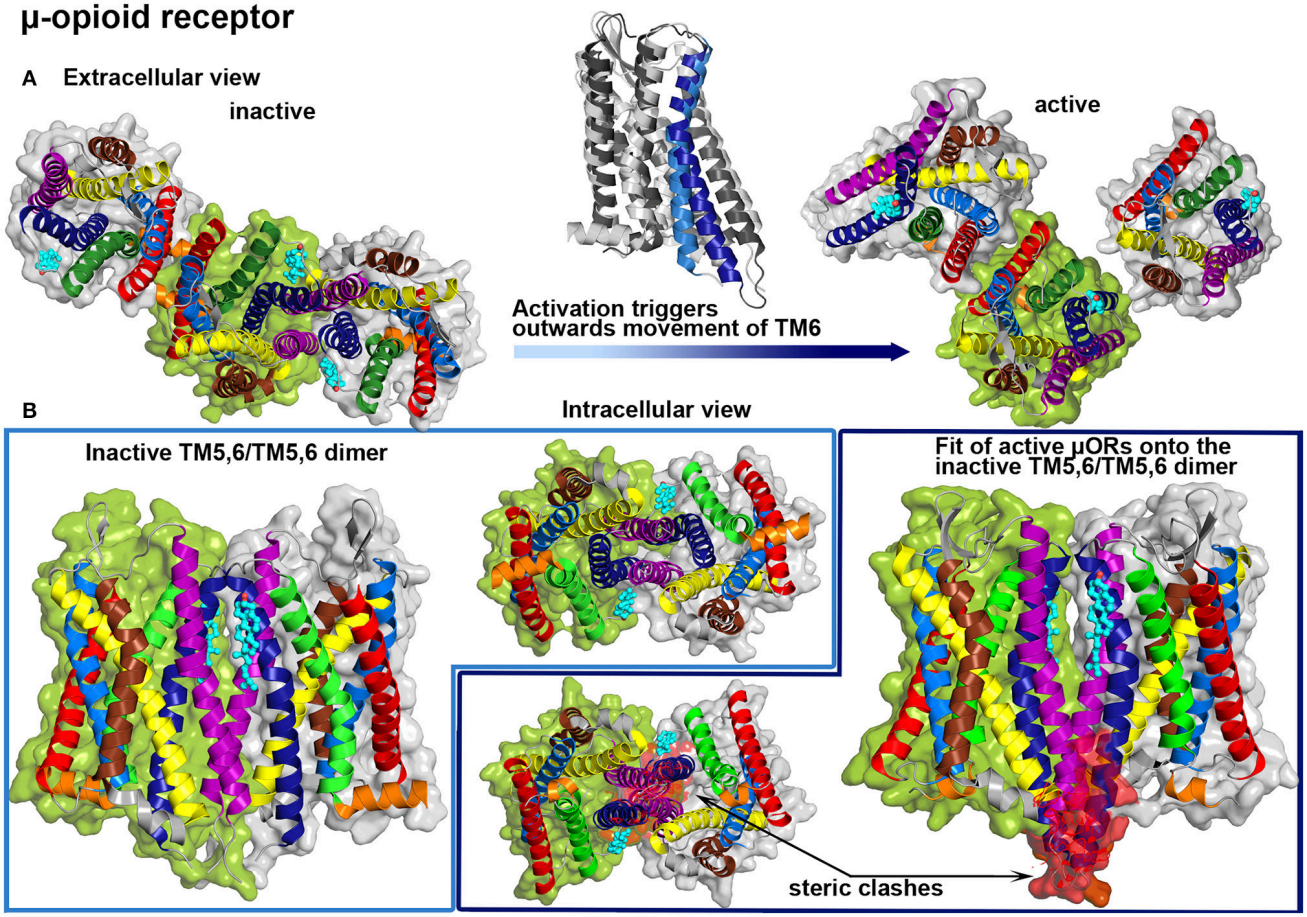
**μ-opioid (μOR) crystal structures**. Color Code: TM1: red, TM2: light blue, TM3: yellow, TM4: brown, TM5: purple, TM6: dark blue, TM7: green, H8: orange, cholesterol: teal. **(A)** μORs dimer interfaces in crystal structures are shown from the extracellular site: inactive protomers (PDB: 4DKL, Manglik et al., 2012, left) and active receptors (PDB: 5C1M, Huang et al., 2015, right). A structure alignment between the inactive (bright colors) and the active (dark colors) form is shown above the transition arrow. **(B)** Inactive μOR form a symmetric TM5,6/TM5,6 interface that is stabilized by cholesterol (left), while a structure alignment of active μORs onto this dimer interface revealed steric clashes at the intracellular parts of TM5 and TM6 (highlighted in red).

Another prominent member of class A GPCRs is the μ-opioid receptor which was also reported to show cholesterol-regulated activity (Huang et al., 2007; Qiu et al., 2011). By comparing the influence of two different membrane nanodomain disrupting agents, namely simvastatin (reductase that blocks cholesterol synthesis) and D-*threo*-1-phenyl-2-decanoylamino-3-morpholino-1-propanol (DPDMP, inhibitor that blocks glycosphingolipid synthesis), possible regulation mechanisms of cholesterol in receptor function were investigated (Zheng et al., 2012b). DPDMP disrupted membrane nanodomains without reducing the cholesterol content but did not affect G protein coupling to the μ-opioid receptor, whereas treatment with simvastatin impaired receptor signaling. Therefore, it was suggested that cholesterol is not only important to stabilize membrane nanodomains in order to provide a platform for protein partitioning, thus facilitating the coupling between receptors and G proteins. Moreover, direct protein-cholesterol interactions seem to be essential for proper signaling. Several earlier studies suggested that the association of μ-opioid receptors to homo- or heteromultimers with other opiod receptors also plays a role in receptor function (Jordan and Devi, 1999; Li-Wei et al., 2002). Recent crystal structures of the μ-opioid receptor revealed receptor dimers and oligomers with bound cholesterol molecules (Manglik et al., 2012; Huang et al., 2015). The first crystal structure was obtained from antagonist-bound receptors and actually revealed two protein-protein interfaces formed by TM1,TM2,H8/TM1,TM2,H8 or TM5,6/TM5,6 interactions (Manglik et al., 2012) (see Figure 4A). The latter interface appears to be stabilized by cholesterol molecules located at the N-terminal groove between TM6 and TM7 reaching toward TM5 of the associated receptor. Interestingly, the second crystal structure shows the μ-opioid receptor bound to an agonist and coupled to a G protein mimetic camelid antibody fragment, thus resembling a ligand-activated GPCR configuration (Huang et al., 2015) (see Figure 4A). As reported for the former structure, one cholesterol molecule is bound to the groove between TM6 and TM7 and one of the dimeric interfaces is mainly constructed by symmetric interactions involving TM1, TM2 and H8. On the other hand, due to the activation-related outwards movement of the intracellular part of TM6, the compact TM5,6/TM5,6 dimer, as it was observed for inactive receptors, could not be formed by active receptors (see Figure 4B).

These findings again illustrate, that dimer conformations of GPCRs differ between active and inactive forms and additionally allow to speculate that specific cholesterol intercalation may contribute to the regulation of GPCR activation. This regulation may be especially important in case of receptors that activate constitutively. Constitutive activation was proposed for several GPCRs if the “ionic lock”-salt bridge between an arginine, located in the conserved intracellular DRY motif on TM3, and negatively charged residues on TM6 is missing (Schneider et al., 2010).

#### 5.1.1. Cholesterol intercalation in GPCR crystal structures

**Figure 5:**
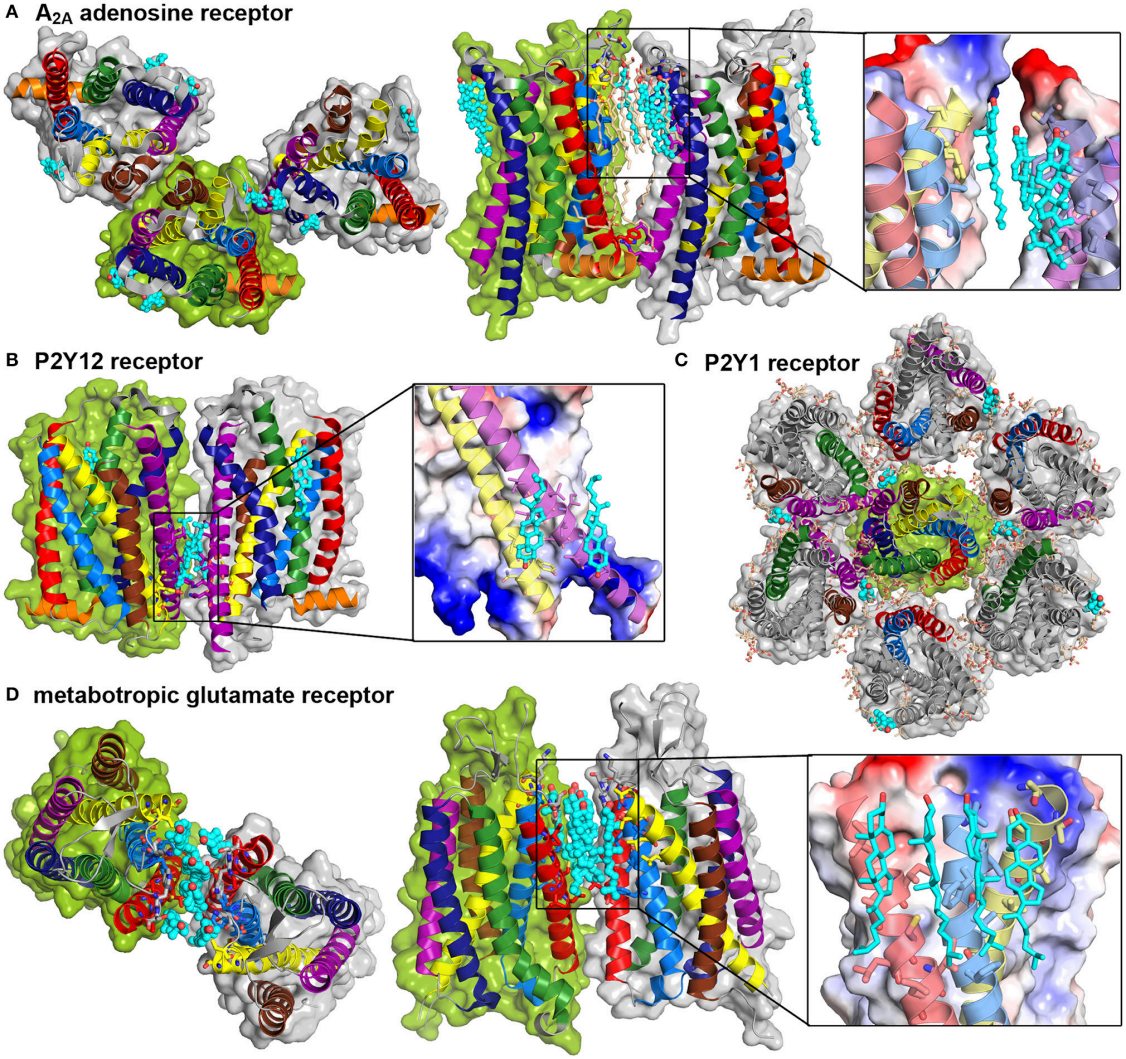
**GPCR crystal structures show cholesterol intercalation between protomers**. Crystal structures and packing of four different GPCRs. Color Code: TM1: red, TM2: light blue, TM3: yellow, TM4: brown, TM5: purple, TM6: dark blue, TM7: green, H8: orange, cholesterol: teal. Detailed cholesterol binding sites are depicted in a combination of surface, colored according to charge (red: negative, blue: positive) and cartoon representation. **(A)** A_2*A*_ adenosine receptor dimers (PDB: 4EIY, Liu et al., 2012b) are shown from the extracellular site and in a site view, additional lipids are shown in light orange. **(B)** P2Y12 receptor dimer (PDB: 4NTJ, Zhang et al., 2014) in a site view, **(C)** P2Y1 receptor heptamer (PDB: 4XNV, Zhang et al., 2015) from the extracellular site, **(D)** Metabotrobic glutamate receptor dimer (PDB: 4OR2, Wu et al., 2014) from the extracellular site and in a site view.

In regard to the continuously growing number of available GPCR crystal structures, the requirement of cholesterol during the crystallization trial is reported with increasing regularity (Salom et al., 2013; Gimpl, 2016). Some of these GPCR structures were actually solved as receptor dimers or oligomers and showed cholesterol intercalation between protomers. The crystal structure of the A_2*A*_ adenosine receptor showed an asymmetric TM1-TM3/TM5,6 interface (Liu et al., 2012b) (see Figure 5A). In total three cholesterol and 14 single chain lipid molecules, mainly from the extracellular leaflet, are present between the protomers. One cholesterol molecule is bound to a groove between TM2 and TM3, which is further stabilized by a hydrogen bond between cholesterol and the extracellular loop between these helices. The other two sterols are closely packed to TM6 reaching into grooves between either TM5 and TM6 or TM6 and TM7. The crystal packing additionally shows a symmetric TM4,5/TM4,5 dimer interface without any intercalating cholesterol molecules, however a small number of lipids reaching into the interprotein clefts. The interplay between the A_2*A*_ adenosine receptor and cholesterol was analyzed earlier with biochemical techniques and revealed the requirement of cholesterol for proper G protein coupling (Zezula and Freissmuth, 2008). Further computational studies suggested that binding of cholesterol molecules to TM2 markedly stabilized the receptor conformation (Lyman et al., 2009).

The above discussed P2Y12 receptor was recently investigated via protein crystallography. The resolved dimer structure involved TM5 and TM6 contacts (Zhang et al., 2014). This dimer interface is further stabilized by bound cholesterol from the lower intracellular leaflet between TM3 and TM5 of each receptor (see Figure 5B). Another cholesterol molecule from the extracellular leaflet was found to bind between TM1 and TM7, thus not contributing to the association interface. In addition, a symmetric TM4,5/TM4,5 interface could be resolved without bound sterols. The related receptor P2Y1 was as well crystallized with one cholesterol bound to the extracellular groove between TM4 and TM5 (Zhang et al., 2015). The crystal packing reveals a multimeric superstructure where one TM4,5-bound cholesterol is in close contact to TM7 of one neighboring receptor and close to TM1 and TM2 of another neighbor (see Figure 5C). In addition to the cholesterol molecules, several lipids fill up gaps between the protomers.

As already mentioned, oligomerization is overall accepted to be essential for class C GPCR function (Kniazeff et al., 2011). The most famous member of this subfamily is the metabotropic glutamate receptor mGluR whose transmembrane domain was recently resolved (Wu et al., 2014). Here, six cholesterol molecules are intercalated between the receptor protomers with close contacts to extracellular, mainly hydrophobic residues of TM1, TM2, and TM3 (see Figure 5D).

In summary, cholesterol appears as a key player in regulating GPCR function. Cholesterol has the ability to adjust membrane properties and to directly interact with receptor monomers and oligomers. Especially direct interactions between GPCRs and cholesterol are proposed to offer complex regulation mechanisms of receptor function: Cholesterol-binding to specific parts of the receptor surface promotes distinct configurations either by occupying parts of the protein, thus disabling certain areas from contributing to a dimer interface, or by intercalating between protomers in order to stabilize certain quaternary structures.

### 5.2. Polyunsaturated fatty acid chains

Another membrane component reported to be involved in GPCR regulation is the polyunsaturated omega-3 fatty acid docosahexaenoic (DHA, 22:6n-3). Membranes with increased concentrations of DHA show low lipid order (Mitchell and Litman, 1998). This low order can be reasoned by the high conformational flexibility of DHA chains due to their large number of unsaturated hydrocarbons which allow for conformational adaptations of the membrane without significant energetic penalty (Gawrisch et al., 2008). Reconstitution of rhodopsin into membranes with high DHA levels revealed that the presence of DHA in close vicinity of the receptor accelerates the formation of the active MII state and coupling to transducin (Niu et al., 2004). In turn, Mitchell *et al*. reported an inhibitory effect of increased levels of cholesterol and phospholipid acyl chain saturation on rhodopsin function (Mitchell et al., 2003). In addition to internal free energy changes, it was proposed that the differences in shape between the inactive MI and the MII conformation imply different free energy contributions arising from the work against the lateral pressure from the surrounding lipids (Gawrisch et al., 2008). Membranes containing more polyunsaturated fatty acids typically exhibit a decreased thickness (for equally long fatty acid chains) and an increased area per lipid. This structural change as well as the difference in order will be accompanied by changes in the lateral pressure profile of the membrane and thus modulate the equilibrium between different receptor states that differ in their cross-sectional area.

Apart from DHA-related differences in the lateral pressure acting on the receptor, an altered membrane curvature was suggested to modulate the GPCR function (Escribá et al., 2007). The lipid composition of the retinal rod outer segment membrane, where rhodopsin is naturally abundant, contains up to 50% of DHA lipids and it was shown that these lipids tend to induce a negative spontaneous curvature, thus facilitating the elongation of the receptor upon activation (Feller and Gawrisch, 2005).

Another hypothesis concerning the preferred solvation of rhodopsin in DHA-rich membranes was contributed from a computational study. It was suggested that the entropic penalty for lipid-protein contacts is significantly smaller for unsaturated chains as compared to saturated hydrocarbon tails (Grossfield et al., 2006b). The reduction of entropic penalty can be reasoned by the increased flexibility and energetic degeneracy of DHA chains as compared to saturated lipids. Even tightly bound DHA chains at the protein surface were reported to reorient at similar rates as lipids distal of the protein (Feller et al., 2003; Grossfield et al., 2006b). Also direct binding of DHA chains to the receptor surface was reported for rhodopsin. It was suggested that especially π-π interactions between the acyl chain double bonds and the aromatic side chains of the receptor surface stabilize the protein-lipid interactions (Gawrisch et al., 2008).

Additionally, due to their high flexibility, DHA chains can easily penetrate or adopt to the structured surface of the TM portion of the receptor with lower energy costs as compared to saturated side chains (Feller et al., 2003). Using MD simulations, it was shown that DHA associates to a small number of distinct regions on rhodopsin (mainly grooves between helices) and that binding of DHA weakens local helix-helix interactions which may facilitate the transition to the MII state (Grossfield et al., 2006a).

A recent study analyzed the effect of DHA chains on receptor oligomerization with a combination of multiscale computer modeling and BRET experiments (Guixà-González et al., 2016). It was shown that DHA improves the oligomerization kinetics of adenosine A_2*A*_ and dopamine D_2_ receptors. In coarse-grained simulations, it was observed that DHA-rich lipids cluster around both receptors and that high levels of DHA enhance protein heteromerization. However, the amount of oligomers was not increased by DHA lipids in the BRET experiments. Therefore, the authors concluded a purely kinetic modulation of the oligomerization. Possible explanations for these observations are increased lateral diffusion rates in DHA-rich membranes and a possible lipid phase segregation into DHA-rich and DHA-poor domains. It was suggested that the redistribution of other membrane components around DHA leads to partioning of DHA into DHA-rich domains (Pitman et al., 2005).

The aggregation of proteins may thus be accelerated due to membrane-driven colocalization of DHA-coated proteins, hence the oligomerization kinetics of DHA-coated proteins do not purely depend on protein-protein interactions alone. Moreover, additional attraction between DHA chains improves the aggregation kinetics (Guixà-González et al., 2016).

## 6. Palmitoylation of GPCRs

Besides interactions between proteins and membrane components, lipids can also be covalently bound to GPCRs. Due to a post-translational modification called palmitoylation, the saturated fatty acid palmitic acid (16 carbons) is usually added to carboxyl-terminal cysteine residues via a thioester-type bond (Chini and Parenti, 2009). It was reported that GPCRs can be mono-, bis- or even tris-palmitoylated and that this lipid modification is reversible as well as adjustable, allowing thereby regulation of GPCR function (Qanbar and Bouvier, 2003). However, this modulation of GPCR function by means of palmitoylation is not a common feature of all GPCRs, given that palmitoylation was reported to be either independent or dependent on ligand binding (Chini and Parenti, 2009), and also the effect on GPCR function was reported as either significant (Blanpain et al., 2001) or only small (Ponimaskin et al., 2002).

The serotonin 5-HT_1*A*_ receptor was shown to be naturally acylated at the two conserved cysteine residues 417 and 420 (Papoucheva et al., 2004). The palmitoylation was found to be independent of agonist stimulation and further to occur early after receptor synthesis. Prevention of the post-translational modifications by mutating the carboxyl-terminal cysteines, weakened or even abolished coupling between receptors and G proteins, thus reducing the native inhibition of forskolin-promoted cAMP formation upon agonist stimulation. These findings suggest that acylation stabilizes a specific conformation of the carboxyl-terminal part to facilitate G protein coupling, or that the attached palmitic acids regulate the receptors trafficking and localization into membrane nanodomains, which was discussed before as important for proper signaling (Papoucheva et al., 2004). Fluorescence and gradient centrifugation studies on the serotonin 5-HT_1*A*_ receptor indeed revealed that native palmitoylated receptors localize mainly in membrane nanodomains, whereas palmitoylation-deficient mutants are considerably less abundant in these raft domains (Renner et al., 2007). The disruption of nanodomains by using methyl-β-cyclodextrin led to reduced localization of wild type 5-HT_1*A*_ receptors and G protein subunits (Renner et al., 2007). Therefore, the palmitoylation-driven colocalization of 5-HT_1*A*_ receptors is considered important for regulating the signaling process (Renner et al., 2007).

Using advanced FRET techniques, the effect of plalmitoylation on 5-HT_1*A*_ receptor oligomers was investigated (Kobe et al., 2008). Wild type acylated receptors showed significantly decreased FRET efficiencies upon agonist stimulation, however co-immunoprecipitation and cross-linking studies revealed a similar amount of oligomers. Consequently, agonist-binding was suggested to induce changes in the oligomeric conformation (into a FRET-negative conformation) rather than dissociation of monomers from protein complexes. In contrast, the FRET efficiency was increased in cells expressing non-acylated receptor mutants and the agonist-induced decrease of FRET efficiency was completely abolished (Kobe et al., 2008). Additionally, cholesterol depletion significantly increased the FRET signal only for wild type receptors, indicating the conversion back into the FRET-positive conformation. These findings for the 5-HT_1*A*_ receptor hint to a mechanism where palmitoylation caters for the compartmentalization of receptors and of receptor oligomers in membrane nanodomains. It further allows for agonist-induced changes of oligomeric conformation either by direct interactions between the palmitic acids and specific membrane components, or by facilitating coupling to G proteins (which are also more abundant in membrane nanodomains). Receptor oligomers located outside of these membrane areas may partly be non-functional but could be “turned on” by palmitoylation-mediated trafficking into the corresponding membrane nanodomains (Kobe et al., 2008). The structure of the related serotonin 5-HT_2*B*_ receptor was recently solved using X-ray crystallography and showed a palmitoylated cysteine (Cys397) on H8 (Wacker et al., 2013). Furthermore, one cholesterol molecule was located in the valley formed between TM1 and H8 with two monoolein molecules in close vicinity.

**Figure 6:**
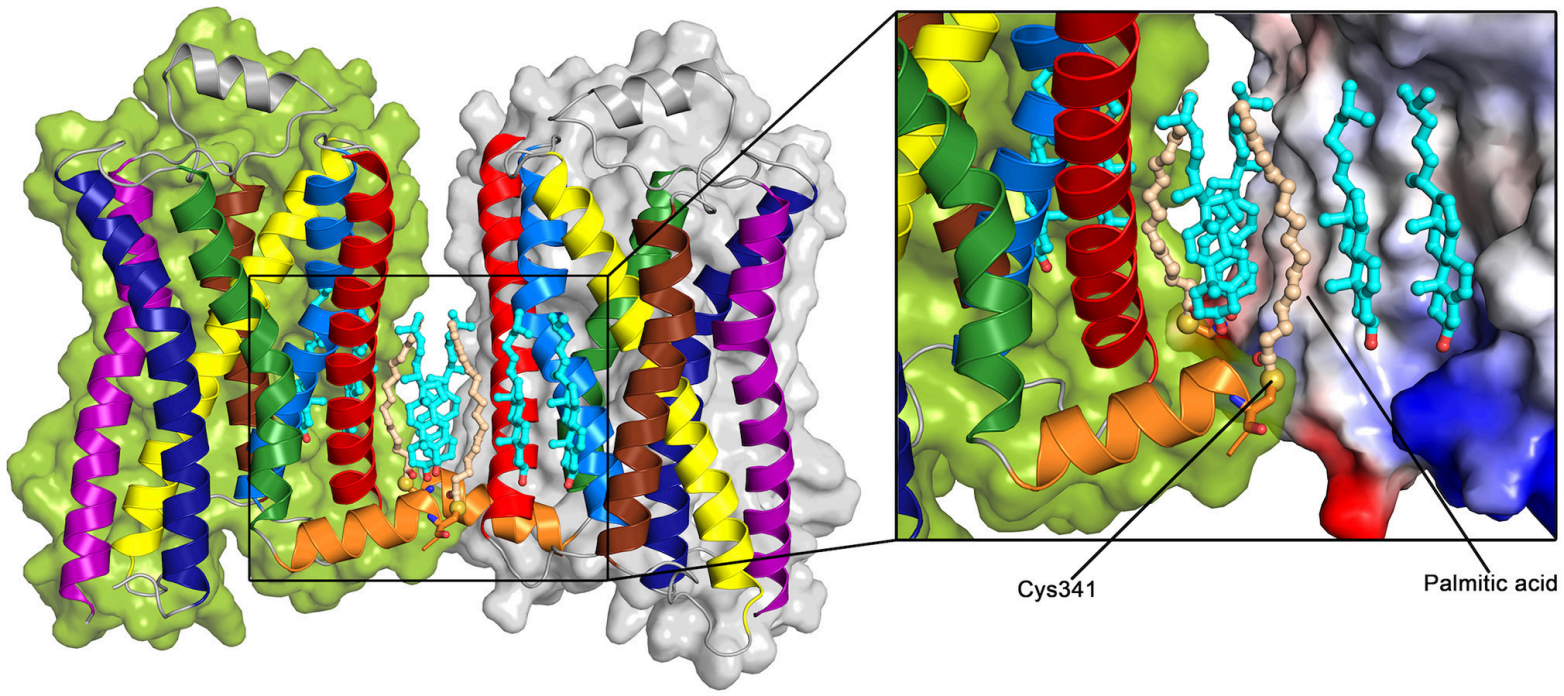
**Palmitoylated β_2_AR dimer (PDB: 2RH1 Cherezov et al., 2007)**. Color Code: TM1: red, TM2: light blue, TM3: yellow, TM4: brown, TM5: purple, TM6: dark blue, TM7: green, H8: orange, cholesterol: teal, palmitic acid: light orange. The sulfur group of the palmitoylated Cys341 is shown as a yellow sphere. The surface of the receptor (right) is colored according to the charge of the receptor (inset, red: negative, blue: positive).

A crystal structure of a β_2_AR dimer was solved including a palmitoylated cysteine residue on the H8 helix (Cys341) at the dimer interface (Cherezov et al., 2007) (see Figure 6). In addition to the palmitic acids, six cholesterol molecules are involved in the symmertic TM1,H8/TM1,H8 dimer interface. Two of these cholesterol molecules appear to be stabilized by interactions with the palmitic acids. Overall, more than 70% of the total buried surface area (515 Å^2^) at the interface is mediated by lipids, and direct protein-protein interactions are only observed between the charged lysine at position 60 on TM1 and the glutamic acid at position 338 on H8.

This dimer structure as well as previous studies on the importance of membrane cholesterol for β_2_AR-related signaling suggest the possibility of a specific modulation of β_2_AR function by membrane components. While the β_2_AR couples to G_*s*_ and G_*i*_ proteins with similar rates in living cells, depletion of cholesterol revealed an increased coupling to G_*s*_ proteins (Xiang et al., 2002). Additionally, G_*s*_ protein coupling was reported to occur to receptor monomers (Whorton et al., 2007). The palmitoylation-mediated cholesterol recruitment possibly facilitates receptor dimerization and thus may shift the coupling preference over to G_*i*_ proteins due to the formation of a specific quaternary structure of the β_2_AR.

The influence of interactions between covalently attached palmitic acids and cholesterol on dimerization were also addressed for the μ-opioid receptor (Zheng et al., 2012a). In constrast to typical palmitoylation sites at the carboxyl-terminus, cysteine 170 on TM3 was identified as the acylation site using immunoblotting techniques. Performing colocalization, co-immunoprecipitation and FRET analyses revealed that wild type receptors oligomerize more efficiently as compared to populations consisting of wild type receptors and mutants (C170A) or exclusively mutated receptors. Additionally, a positive correlation between G protein coupling and receptor palmitoylation was observed suggesting that reduced receptor dimerization decreased G protein coupling. Cholesterol was found to be more frequently associated with receptor complexes formed by wild type receptors hinting to the recruitment of cholesterol by palmitic acid. The importance of associated cholesterol was further analyzed using FRET methods and it was reported that the absence of cholesterol decreased wild type receptor oligomerization and further G protein coupling. To validate these results the influence of a palmitoylation inhibitor (2-BP) was tested. It was indeed observed that upon 2-BP treatment both, homodimerization and cholesterol association were reduced to a similar level as for systems expressing the C170A mutants. In addition, a computational homology model based on a crystal structure of the β_2_AR (Rasmussen et al., 2007) was produced for the μ-opioid receptor to further analyze the suggested the homodimer. Based on Conformational Memories calculations, TM4 was indicated to mainly build up the dimer interface and the palmitic acid attached to TM3 facilitates packing of a cholesterol molecule between TM3 and TM4 of each protomer.

Consequently, similar to the previously discussed β_2_AR dimer, two palmitoylation-recruited cholesterol molecules are directly involved in stabilizing the dimer interface. However, an interesting difference arises from comparing palmitoylation sites and dimeric interfaces between the two described receptors: The β_2_AR appeared to be palmitoylated at the carboxyl-terminal cysteine on H8 which is involved in building up a symmetric TM1,H8/TM1,H8 dimer interface with intercalated cholesterol. In case of the μ-opioid receptor, TM3 carries the palmitoylation site which further mediates cholesterol association to stabilize a symmetric TM3,4/TM3,4 interface. In summary one may speculate that palmitoylation is regulating GPCR function due to the recruitment of cholesterol in order to stabilize a specific dimer interface (Goddard and Watts, 2012b).

## 7. Conclusions

In summary, both the experimental and computational evidence for a significant influence of the lipid environment on GPCR function and organization is overwhelming. Compartmentalization of GPCRs in membrane nanodomains (caveolar and non-caveolar) appears to be essential for numerous GPCR-mediated signaling events. The reasons for this necessity seem to be spread over different factors: Colocalization of GPCRs with other signaling components (mainly G proteins), a decreased hydrophobic mismatch and close vicinity to specific lipids and cholesterol. Especially cholesterol plays a key role in GPCR trafficking, organization and function. Several studies hinted to conformational changes in preexisting dimers and oligomers upon cholesterol depletion (Devost and Zingg, 2004; Paila et al., 2011; Pluhackova et al., 2016). In addition, protein-cholesterol interactions have been reported as essential for efficient GPCR signaling (Nguyen and Taub, 2002, 2003; Zheng et al., 2012b; Jafurulla et al., 2014). Likely, the GPCR dimer equilibrium between both, active and inactive dimers is shifted by the addition/depletion of cholesterol to active/inactive dimers.

Function-competent oligomers are suggested to be stabilized via interactions between their transmembrane helices excluding TM6 (Zheng et al., 2012a; Huang et al., 2015; Pluhackova et al., 2016). In order to regulate the crucial receptor-cholesterol interactions, palmitoylation is revealed to recruit cholesterol to dimeric interfaces thus stabilizing specific quaternary structures (Cherezov et al., 2007; Chini and Parenti, 2009; Zheng et al., 2012a). The influence of specific membrane components can be further analyzed by performing a reconstitution of GPCRs to artifical membrane systems (e.g., GUVs) with controlled properties. Both, protein crystallography or computer-based studies are ideally suited to guide future studies on receptor-receptor and receptor-lipid interactions. Advances in both, experiment and simulation, are expected also to solve the seeming discrepancy between the reported localization of GPCRs in densely packed, probably ordered lipid nano- or microdomains and their association to polyunsaturated and thus disordered lipids.

Besides, GPCR hetero-oligomerization gains increasing interest, since a large number of GPCRs revealed binding cooperativity and heterodimerization-dependent trafficking (Prinster et al., 2005). However, far less structural information is available for hetero-oligomeric complexes as compared to homo-oligomers. In order to further investigate the complex interaction network between GPCRs it is of great importance to determine whether heteromers show a similar lipid-dependent function and organization as homomers. All in all, this review stresses the necessity to carefully include the effect of the lipid nanodomain environment in any study on GPCR structure and function.

## Author contributions

SG and RB developed the concept of this review and wrote the manuscript.

## Funding

This work was funded by the DFG Research Training Group 1962/1, “Dynamic Interactions at Biological Membranes: From Single Molecules to Tissue.”

### Conflict of interest statement

The authors declare that the research was conducted in the absence of any commercial or financial relationships that could be construed as a potential conflict of interest.
